# Altered miRNA Signatures in Follicular Fluid: Insights into Infertility Etiologies

**DOI:** 10.3390/genes16050537

**Published:** 2025-04-30

**Authors:** Cornelia Braicu, Cristina Ciocan, Cecilia Bica, Oana Zanoaga, Laura Ancuta Pop, Stefan Strilciuc, Adelina Staicu, Iulian Goidescu, Daniel Muresan, Mihai Surcel, Ioana Berindan-Neagoe

**Affiliations:** 1 Department of Genomics, MEDFUTURE Institute for Biomedical Research, Iuliu Hațieganu University of Medicine and Pharmacy, 400337 Cluj-Napoca, Romania; cornelia.braicu@umfcluj.ro (C.B.); crisciocan@gmail.com (C.C.); cecilia.bica8@gmail.com (C.B.); zanoaga.oana@gmail.com (O.Z.); laura.pop@umfcluj.ro (L.A.P.); stefan.strilciuc@genomics-center.ro (S.S.); 2 1st Obstetrics and Gynaecology Department, Iuliu Hatieganu University of Medicine and Pharmacy, 400012 Cluj-Napoca, Romaniaiuliangoidescu@gmail.com (I.G.); daniel.muresan@umfcluj.ro (D.M.); 3 Doctoral School, Iuliu Hatieganu University of Medicine and Pharmacy, 400337 Cluj-Napoca, Romania; ioana.neagoe@umfcluj.ro

**Keywords:** infertility, follicular fluid, microarray, microRNA

## Abstract

**Background/Objectives:** Infertility is a reproductive disorder affecting approximately 10–15% of reproductive-age couples worldwide. Recent studies have suggested that miRNAs in follicular fluid may provide insights into reproductive potential and follicle health. This study evaluated the altered profile of miRNAs in the follicular fluid in patients undergoing IVF, considering the underlying etiology of infertility. Among our study participants, we identified four major underlying causes of infertility: polycystic ovary syndrome (PCOS), pelvic inflammatory disease (PID), male factor infertility, and unexplained infertility (UI). **Methods**: This study aimed to assess whether these infertility diagnoses are associated with distinct follicular behaviors and to identify altered miRNA patterns linked to these conditions. Ingenuity Pathway Analysis (IPA) was used to evaluate the impact of the altered miRNA signature on key biological processes. **Results:** The bioinformatics analysis of microarray data revealed altered miRNA patterns in FF for selected subgroups. Compared to healthy controls, 25 differentially expressed miRNAs were identified in PCOS (9 downregulated and 16 overexpressed), 21 in PID (15 downregulated and 6 overexpressed), and 34 in UI (24 downregulated and 10 overexpressed). These altered miRNA signatures indicate a complex interplay with essential signaling pathways, including hormonal regulation and tissue remodeling. **Conclusions**: Our analysis revealed key miRNAs that were differentially expressed across selected groups, indicating their potential as biomarkers for more accurate diagnosis and targeted treatment strategies. These findings provide valuable insights into the molecular mechanisms underlying reproductive disorders and underscore the importance of further research to develop targeted interventions that can enhance patient outcomes.

## 1. Introduction

Infertility is a serious condition impacting approximately 10–15% of couples of reproductive age, with projections suggesting a dramatic increase in prevalence, primarily attributed to the growing trend of delayed childbearing [1]. Given the complexity of the reproductive process and the numerous factors involved, the range of potential issues is vast, with solutions that often fail to resolve the problem efficiently.

Assisted reproductive technology (ART) has emerged as a prevalent intervention for the management of moderate to severe infertility, primarily due to its capacity to surmount various biological impediments [1]. Nevertheless, the efficacy of in vitro fertilization (IVF) warrants thorough re-evaluation, considering that only approximately 7% of retrieved oocytes exhibit the quality required for a successful live birth [2]. A portion of these unfavorable outcomes can be attributed to conditions that disrupt follicular development, while others may result from ovarian hyperstimulation itself [3]. The latter may reveal underlying dysfunctions that would otherwise remain clinically insignificant, thus introducing a new set of challenges in the management of infertility [1,3,4,5], where the pollution has an important impact [6].

Proper endocrine signaling and follicular maturation are essential for developing a mature and competent oocyte. Follicular development depends on robust cooperation between stromal ovarian cells, cumulus cells, and oocytes to facilitate several critical processes, including nutrient exchange (lipid, glucose, and amino acid metabolism), steroidogenesis, oxidative phosphorylation, and the precise regulation of free radicals [7,8], or altered by the presence of different environmental pollutants.

Follicular fluid (FF) is the critical microenvironment where these processes unfold in a precisely regulated temporal sequence. It is rich in diverse metabolites, hormones, growth factors, anticoagulants, electrolytes, reactive oxygen species, antioxidants, and interleukins. Notably, it also contains small regulatory molecules, such as non-coding RNAs, particularly microRNAs (miRNAs).

miRNAs are small, single-stranded RNA molecules, typically about 21–25 nucleotides in length. miRNAs, by targeting messenger RNA (mRNA) transcripts, can either inhibit their translation or promote their degradation [9]. miRNAs have been implicated in various biological processes, including fertility [10,11,12,13,14,15,16]. These transcripts serve as crucial biomarkers, shedding light on follicular development, ovarian function, and reproductive health [13,14,17]. By identifying altered miRNA profiles, novel diagnostic and therapeutic strategies may be developed, leading to improved birth rates in women undergoing fertility treatments [14,18,19,20,21].

This study aimed to evaluate the altered profile of miRNAs in the follicular fluid using microarray technology in patients undergoing IVF, considering the underlying etiology of infertility. Among our study participants, we identified four major underlying causes of infertility: polycystic ovary syndrome (PCOS), pelvic inflammatory disease (PID), male factor infertility, and unexplained infertility (UI), which were assessed in this study to determine whether these infertility diagnoses may be associated with distinct follicular behaviors that may justify an adjusted treatment strategy. Additionally, we analyzed the impact of the altered miRNA signature on key biological processes using Ingenuity Pathway Analysis (IPA). This approach allowed us to gain mechanistic insights into how these miRNAs may influence critical pathways and molecular networks associated with the previously mentioned reproductive disorders.

## 2. Materials and Methods

### 2.1. Population Sample

Our study included 47 women who underwent an in vitro fertilization procedure at the 1st Obstetrics and Gynecology Clinic in Cluj-Napoca (Romania) between April and October 2019 and July and December 2022, respectively. All study participants provided written informed consent before being enrolled in the study, and the research protocol was approved by the “Iuliu Hatieganu” University of Medicine and Pharmacy Ethics Committee (Institutional Review Board (IRB) approval no. 51, 11 March 2019). All study participants completed a physician-administered questionnaire that collected demographic data, socioeconomic status, health status, lifestyle habits, medical history, and potential occupational health hazards. The patients were assigned to groups based on four categories: the PCOS group, the male etiology group, the PID group, and the unexplained infertility group (UI). Patients in the PCOS group were referred based on the Rotterdam criteria, which require the presence of at least two of the following three features: clinical and/or biochemical hyperandrogenism, polycystic ovary morphology, and oligo- or anovulation. Furthermore, conditions presenting with hyperandrogenism were excluded before diagnosing PCOS.

Table 1 presents the distribution of demographic and clinical factors and health-related behaviors among the study participants. They were between 26 and 44 years of age (mean and standard deviation (SD): 35.2 ± 4.8). Most of the participants (76.6%) graduated as faculty. Most women (59.6%) had normal weight, and there were 17 (36.1%) overweight or obese women among our study participants. Regarding the infertility diagnosis, most of the participants were diagnosed with female factor infertility, including “UI” (76.6%). Four fertilized oocytes (range 1–12) were obtained per woman, and two blastocytes (range 0–6) were generated per couple. Twenty (42.6%) women had a pregnancy, of whom one experienced an abortion.

Patients were referred to the PID group based on the diagnosis of mild tubal disease, which was established through ultrasound, hysterosalpingography, or laparoscopy. Patients were enrolled in the unexplained infertility group if they were part of infertile couples with documented normal ovulatory function, tubal patency (as determined by hysterosalpingography or laparoscopy), and a normal semen analysis according to World Health Organization (WHO) criteria. Patients were included in the male etiology infertility group when the partner’s sperm exhibited severe abnormalities in terms of count, morphology, or motility.

Participants were excluded if multiple causes of infertility were identified, if they exhibited mild to moderate male infertility, if they had moderate to severe endometriosis (as classified by the American Society for Reproductive Medicine-ASRM), or if they had a history of pelvic inflammatory disease (PID) that required salpingectomy. For each participant, we abstracted clinical data from the medical records on antral follicle count (AFC) and anti-mullerian hormone (AMH) as indicators of ovarian reserve, hormone levels, response to ovarian stimulation (mature follicles, number of oocytes retrieved, Follicular Output Rate (FORT), the follicle–oocyte index (FOI), and thickness of the endometrial mucosa), and IVF endpoints (number of embryos, pregnancy, abortion, and live births). The three patients with confirmed endometriosis were identified during the clinical evaluation and were excluded from the analyzed cohort to avoid introducing confounding variables that could affect the follicular fluid miRNA profile.

### 2.2. Biological Sample Collection and Clinical Protocol

We collected urine, blood, ovarian follicular fluid, and endometrial flushing fluid samples from our study participants to test for metals and to perform genetic analysis. All biospecimens were immediately stored at either −20 °C for metal analysis or −80 °C for genetic analyses.

The clinical protocol has been thoroughly detailed in our previous publication [22]. In summary, all participants first underwent a baseline infertility assessment, after which they were allocated to a tailored controlled ovarian stimulation protocol, taking into account their individual phenotype, infertility profile, reproductive history, and personal preferences. Either a long protocol (ovarian downregulation with agonist gonadotropin-releasing hormone (GnRH)) or an antagonist GnRH protocol was implemented. The starting gonadotropin doses were adjusted for patient individual factors (e.g., age, body mass index (BMI), reproductive history, etc.). Transvaginal ultrasound and serum estradiol levels were used to monitor the ovarian stimulation, starting four/five days after initiation, and the gonadotropin doses were adjusted according to the ovarian response. Then, choriogonadotropin-α (hCG) was administered for final oocyte maturation. At 34–38 h after hCG administration, the transvaginal follicle puncture was performed to retrieve the oocytes. Retrieved oocytes in metaphase II arrest were fertilized using fresh sperm from the male partner or by intracytoplasmic sperm injection (ICSI) in cases with male pathology. Three to five days after the oocyte retrieval, one to two embryos were transferred into the uterus. A pregnancy was biochemically confirmed by serum β hCG measurement (β hCG > 20 mIU/mL), and a “clinical pregnancy” was confirmed two weeks later by ultrasound examination when one or more gestational sacs were visualized in the uterus [23]. Each study participant’s pregnancy was monitored by an obstetrician who later reported either a spontaneous abortion or a live birth.

### 2.3. miRNA Analysis

The altered miRNA pattern was analyzed using 100 ng of total RNA per sample. The Agilent microRNA Spike-In kit was used to hybridize samples, while labeling was performed with the miRNA Complete Labeling and Hyb Kit (Agilent, Santa Clara, USA). To minimize the risk of artifacts, a purification step was implemented using Micro Bio-Spin 6 spin columns from Biorad, followed by desiccation in a vacuum centrifuge and resuspension of the pellet in 18 μL of RNase-free, microbiologically pure water. Hybridization followed the manufacturer’s guidelines, with slides (Agilent SurePrint Human miRNA v21.0 microarray, G4872A) left in the hybridization oven for 20 h at 55 °C. After washing, the slides were scanned using an Agilent Microarray Scanner.

### 2.4. Bioinformatic and Statistical Analysis

After scanning the microarray slides, the Feature Extraction software (Agilent Technologies, version 12.1, Agilent, Santa Clara, CA, USA) was used to analyze the images and convert them to numeric expression values. The individual files were fed into the Agilent GeneSpring GX program (Agilent Technologies) for data normalization, which was conducted using the quantile algorithm. No baseline transformation was performed. Entities were initially filtered based on their flag values, keeping them acceptable on the “detected” ones. Differentially expressed genes were selected using the “Filter on Volcano Plot” analysis and *t*-test unpaired, with a fold change (FC) of 1.5 and a *p*-value < 0.05 for comparison in study participants with UI, PID, and PCOS diagnoses versus a group of study participants with male factor infertility diagnosis. Ingenuity Pathway Analysis (IPA, Redwood, CA, USA), a data mining software, was employed to conduct additional analysis of the altered miRNA pattern in patients with UI, PID, and PCOS diagnoses.

### 2.5. TGFβ1 and TNFα Protein Serum Quantification Using ELISA

The expression levels of TGFβ1 in the cell culture medium were detected by ELISA using the Human TGFβ1 DuoSet ELISA (R&D System, cat no. DY240, Minneapolis, MI, USA), and for TNF-α, the Human TNF-α DuoSet ELISA (R&D System, cat no. D210) was used along with the DuoSet Ancillary Reagent Kit 2 (R&D Systems, cat no. DY008), as described in detail in our previous work [24].

## 3. Results

**Study Cohort.** The microarray study cohort was composed of participants diagnosed with polycystic ovary syndrome (PCOS, n = 8), pelvic inflammatory disease (PID, n = 7), or unexplained infertility (UI, n = 3). The control group comprised participants whose infertility was attributed to a male factor (n = 3), ensuring that the female participants had no underlying reproductive disorders (Table 2).

**Figure 1:**
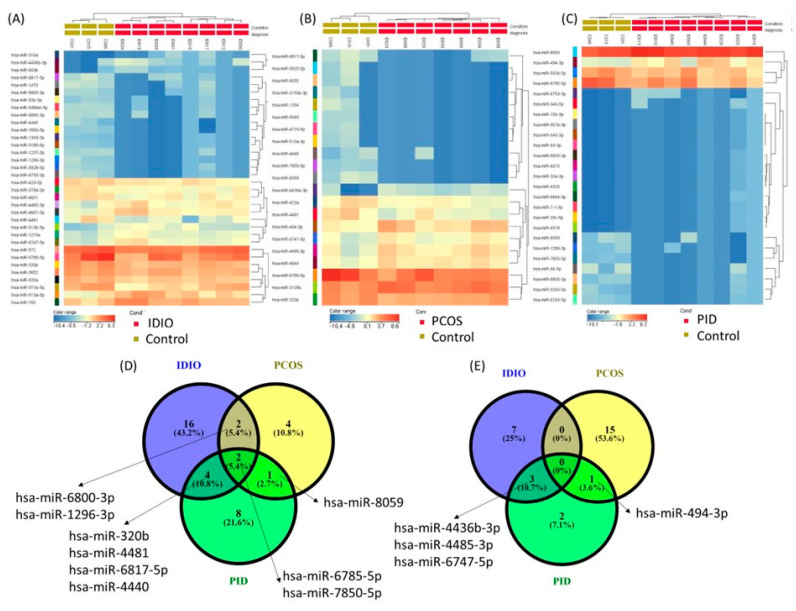
Altered miRNA patterns in study participants with PCOS, PID, and UI diagnoses versus a group of study participants with male factor infertility diagnosis; red or blue colors indicate differentially up- or downregulated miRNAs, respectively. (**A**) Heatmap representation of results in patients with PCOS infertility diagnosis; (**B**) heatmap representation of results in patients with PID infertility diagnosis; (**C**) heatmap representation of results in patients with UI infertility diagnosis; (**D**) Venn diagram for the downregulated miRNAs for the analyzed groups; and (**E**) Venn diagram for the overexpressed miRNAs for the analyzed groups.

**miRNA altered pattern in PCOS, PID, and UI.** The bioinformatics analysis results, considering as a cut-off value *p* < 0.05 and FC ± 1.5, showed 25 differently expressed miRNAs, of which 9 were underexpressed and 16 were overexpressed in patients with PCOS infertility diagnosis; 21 differently expressed miRNAs, of which 15 were underexpressed and 6 were overexpressed in patients with a PID diagnosis; and 34 differently expressed miRNAs, of which 24 were underexpressed and 10 were overexpressed in patients with a UI diagnosis. A list of miRNAs with an altered expression level, expressed as fold change (FC) and *p*-value in study participants, is displayed in Table 3. Figure 1 shows a heatmap representation of results in study participants with UI, PCOS, and PID diagnoses versus a group of study participants with male factor infertility diagnosis.

**IPA analysis of altered miRNA in PCOS, PID, and UI diagnoses.** The IPA analysis examined altered miRNA patterns within the investigated groups, summarized the biological significance, and identified molecular interactions between altered miRNAs and their target genes. Table 4 highlights the common associations between diseases and disorders, as well as molecular and cellular functions, based on altered miRNA patterns in patients with PCOS, PID, or UI.

The analysis indicated that all three groups shared significant miRNA-related changes associated with organ injury and abnormalities, inflammatory diseases, and cancer. At the molecular and cellular levels, common alterations were observed in cellular movement, development, growth, proliferation, cell death, and survival. These shared miRNA-driven changes suggest partially overlapping pathways and mechanisms underlying these infertility-related conditions.

**Figure 2:**
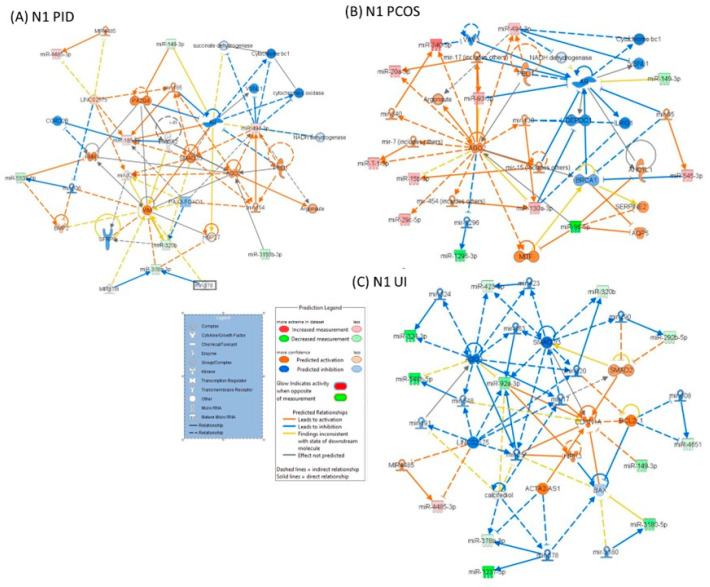
IPA network analysis of altered miRNA patterns in patients with infertility diagnosis versus the control group: (**A**) patients with PCOS infertility diagnosis, (**B**) patients with PID infertility diagnosis, and (**C**) patients with UI diagnosis. The network diagram includes miRNA and edge (gene relationship) symbols. Red indicates upregulated genes, and green indicates the downregulation of miRNA. Activation and inhibition predictions are denoted by orange and blue, respectively. Molecules associated with miRNAs for which no directional change prediction could be determined are indicated in white. Lines connecting molecules and miRNAs represent various relationships, as outlined in the legend.

The IPA analysis showed the key biological pathways and functions disrupted in each analyzed group (Table 4). In PID, the altered miRNA pattern integrates into a network containing key molecules related to Cellular Movement, Organ Injury and Abnormalities, and Reproductive System Disease (Figure 2A). In the PCOS group, the leading network involves cellular movement, organismal injury, and reproductive system disease (Figure 2B). In the UI group of patients, the primary networks were associated with gene expression, neurological diseases, organ injury, cellular development, growth, and movement (Figure 2C).

**Figure 3:**
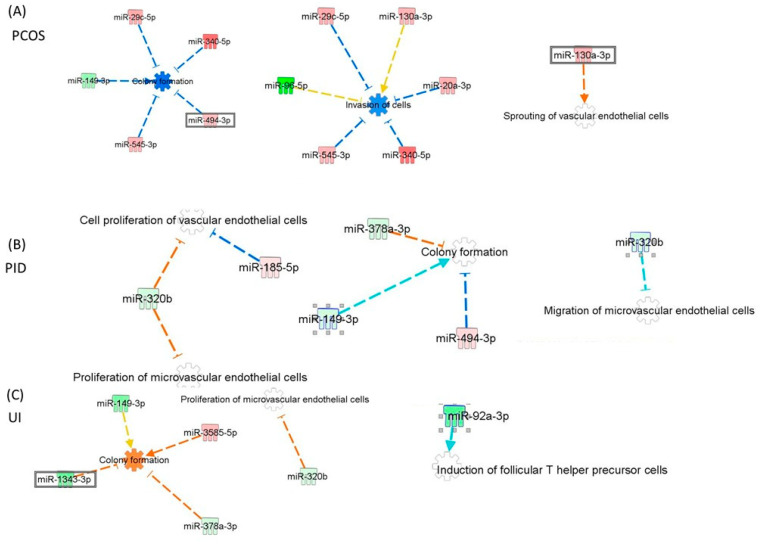
IPA network analysis of altered miRNA patterns in study participants with UI PCOS and PID diagnoses versus a group of study participants with male factor infertility diagnosis: (**A**) patients with PCOS diagnosis, (**B**) patients with PID diagnosis, (**C**) patients with UI diagnosis. miRNA and edge (gene relationship) symbols are shown along with the network diagram. Red shows upregulated genes, and green indicates downregulation of miRNA.

miRNAs may influence cellular functions crucial for reproductive health. Figure 3 shows miRNA interactions and their roles in critical cellular processes such as endothelial cell proliferation, migration, invasion, and sprouting; colony formation; and immune cell differentiation. miR-149-3p and miR-320b, which are involved in endothelial cell proliferation, could play a role in reproductive tissue vascularization. For PID, miRNAs are linked to immune response and cell invasion. miR-320b and miR-185-5p regulate the proliferation of vascular and microvascular endothelial cells, which is crucial for new blood vessel formation, to repair tissue damaged by inflammation. Additionally, miR-320b plays a crucial role in endothelial cell migration, tissue regeneration, and immune cell recruitment, processes essential for the inflammatory response characteristic of PID. The involvement of miRNAs such as miR-378a-3p, miR-149-3p, and miR-494-3p in colony formation further supports cell survival and repair mechanisms, highlighting the importance of these miRNAs in managing inflammation and facilitating healing in PID (Figure 3A). In the case of PCOS diagnosis, miRNAs such as miR-29c-5p, miR-340-5p, miR-378a-3p, and miR-494-3p, which are involved in colony formation and cellular proliferation, may contribute to the excessive follicular growth and abnormal ovulation associated with this condition. Additionally, miR-130a-3p’s role in vascular endothelial cell sprouting could influence ovarian angiogenesis, affecting follicle development and potentially exacerbating the hormonal imbalance in PCOS (Figure 3B). Similarly, miRNAs associated with cell invasion and migration (e.g., miR-96-5p, miR-340-5p) might impact processes such as embryo implantation or uterine receptivity, thus leading to infertility in UI patients (Figure 3C).

**Figure 4:**
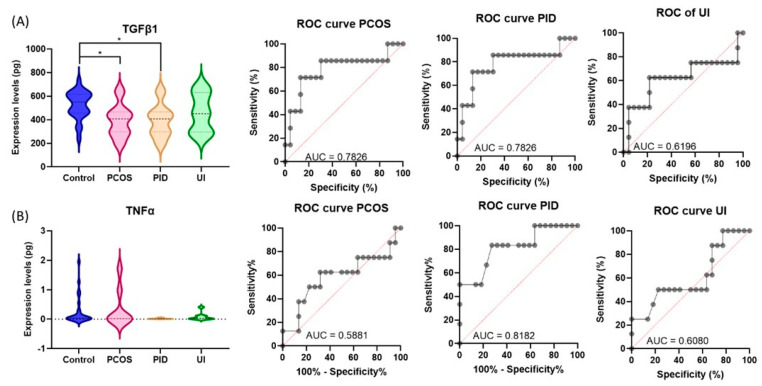
TGFβ1 (**A**)and TNFα (**B**) protein serum quantification using ELISA for PID, PCOS, and UI (* *p* < 0.05, statistically significant difference between the groups being compared). Serum quantification of TGFβ1 (**A**) and TNFα (**B**) using ELISA in patients with PID, PCOS, and UI. Statistically significant differences between the compared groups are indicated (* *p* < 0.05).

**Protein serum quantification using ELISA for PID, PCOS, and UI.** TGFβ1 and TNFα were selected for validation due to their key roles in inflammation, immune response, and tissue remodeling, processes central to PID, PCOS, and UI; the results are shown in Figure 4A. The analysis showed decreased levels of TGFβ1 in patients with PCOS and PID but no alteration in the UI group versus a group of study participants with male factor infertility. In the case of TNFα serum protein, there was no significant alteration among the investigated patient groups (Figure 4B). Additional graphical representation of ROC curves reveals a low specificity as a biomarker for most of the evaluated groups.

## 4. Discussion

Recent studies have indicated that miRNAs are abundant in follicular fluid, playing crucial roles in reproductive processes and emphasizing specific miRNA patterns in UI, PCOS, and PID [25]. Consistent with the findings of Sang et al. [26], our study identified differential miRNA expression patterns in women with PCOS compared to a group of participants with male factor infertility diagnosis. Our data suggest that miRNAs may play a critical role as regulators in reproductive diseases. The IPA analysis showed that their altered expression may contribute to the onset and progression of these conditions by disrupting critical biological pathways. When analyzing each infertility category, we identified a distinct pattern that could characterize the group. Also, we could emphasize common features of the altered miRNA interaction with argonaute proteins or those related to colony formation or the regulation of vascular cell endothelial functions. Endothelial cell function is critical in maintaining reproductive system homeostasis [27]. 

The dysregulation of miRNAs can affect the expression of their target genes, resulting in significant alterations in vital cellular pathways that are involved in reproductive disorders. This disruption can induce inflammation or alter processes such as cellular growth and tissue remodeling, which are critical in the development and progression of conditions that may impair follicular development, such as UI, PID, and PCOS, as shown by our analysis. PCOS is a complex and controversial disorder, typically characterized by anovulation, hyperandrogenism, and altered ovarian morphology [28,29]. Beyond this basic definition, PCOS is associated with a wide and diverse range of pathogenetic mechanisms, including molecular abnormalities related to neuroendocrine disruption, genes encoding signaling components involved in steroidogenesis, steroid hormone action, gonadotropin action and regulation, insulin action and secretion, energy metabolism, chronic inflammation, and oxidative stress [28]. These diverse factors collectively contribute to the complexity of the condition and help explain why this simplistic definition may mask a variety of phenotypes with clinically distinct behaviors. In this regard, new tools that identify altered pathogenetic pathways are essential for accurately addressing each phenotype [28]. Recent data are already available that support the role of miRNAs in elucidating the pathogenic mechanisms [29,30,31]. An aberrant miRNA-mRNA regulatory network was identified as being linked to markers of insulin sensitivity and inflammation, highlighting its potential as a key contributor to disease pathophysiology [30,32].

PID is regarded as a gynecologic condition in which the fallopian tubes are primarily affected, leading to compromised sperm and egg transportation. Additionally, it serves as a source of toxins that negatively impact the endometrium. In clinical practice, the impact of PID on ovarian function remains uncertain. Consequently, except in cases where a significant volume of hydrosalpinx is present, which may alter the implantation process, the removal of the fallopian tubes is typically considered to have no clinically significant effect on the success rate of IVF. Our results partially corroborate the existing data; however, several alterations in the follicular fluid can be identified in patients with PID, even in cases of minor tubal pathology [33,34]. UI is a distinctive diagnosis, essentially an exclusionary one, made when all major causes—such as tubal, male, and anovulatory factors—have been ruled out. It assumes the underlying cause is subtle and may reside within the ovaries or elsewhere outside the gonads. Analyzing follicular fluid is the most logical approach to identifying phenotypes that exhibit follicular disturbances [35,36].

Argonaute proteins, particularly AGO2, are crucial components of the RNA-induced silencing complex (RISC), which regulates gene expression through RNA interference [37]. Aberrations in RNA interference pathways, including those involving AGO proteins, can impact gene expression in cell proliferation and survival [37,38], which is observed to be targeted by altered miRNAs in UI and PCOS. This dysregulation can contribute to the abnormal growth of ovarian tissues [38]. The dysregulation of AGO2 may lead to the aberrant expression of target genes, contributing to chronic inflammation and tissue damage.

The dysregulation of SMAD2/3 could contribute to abnormalities in key biological processes, potentially leading to impaired fertility. SMAD2/3 is part of the TGFβ signaling pathway and contributes to changes in the extracellular matrix and tissue remodeling [39,40]. This component is also the core of the N1 networks in UI. In UI infertility, alterations in SMAD2 and SMAD2/3 signaling could potentially impact reproductive functions. These molecules are critical in mediating the effects of TGFβ on ovarian function, folliculogenesis, and the endometrial environment. 

Androgen Receptor (AR), known for its role in male reproductive physiology, has been shown to play a role in female reproductive disorders, as well [41]. Its involvement in PCOS and PID suggests that hormonal signaling, particularly androgen signaling, may influence the inflammatory processes in the pelvic region [42]. This could impact the progression of PCOS and PID, or the body’s response to the infection that triggers the disease [41,42].

Vimentin (VIM), a marker of epithelial–mesenchymal transition (EMT), is often associated with alterations in the extracellular matrix and tissue remodeling [43]. Therefore, the EMT and the associated changes in cellular architecture could contribute to structural alterations in the reproductive organs, affecting the tissue structure (e.g., scar tissue, a common consequence of chronic PID). 

Our data demonstrate a complex interaction between miRNA regulation, hormonal signaling, tissue remodeling, and immune response modulation. In this context, the study of miRNAs presents significant potential as a valuable tool for understanding both clinically evident and subclinical conditions associated with infertility. Beyond describing these conditions, miRNA research could provide crucial insights into the underlying molecular mechanisms driving infertility. This deeper understanding would not only enhance our knowledge of the pathophysiology of infertility but also inform the development of more targeted medical approaches for effective diagnosis and treatment. However, the findings of this study, while insightful, reveal limitations inherent to prevailing paradigms in infertility research and treatment. The traditional concept of infertility has largely focused on mechanical factors, such as fallopian tube integrity, sperm motility, and the peritoneal environment, while often neglecting the pivotal role of folliculogenesis. IVF has traditionally been used to circumvent these issues, with a focus on follicular development. This shift in clinical practice has led to categorizing patients based on their follicular response to ovarian stimulation, distinguishing them as normal responders, poor responders, and hyper-responders. While this classification has facilitated improvements in the number of oocytes retrieved, it has not been as effective in enhancing oocyte quality. 

This study is one of the few that have analyzed the altered miRNA profiles in follicular fluid within a Romanian subpopulation. Our findings provide a foundation for more extensive future research to enhance IVF success rates among Romanian patients. However, our study has several limitations. The participants were recruited from a single IVF treatment center, which may limit the generalizability of the findings to all Romanian IVF patients. Additionally, the study was limited by a small sample size and the absence of randomization, which may have affected the robustness of the results.

## 5. Conclusions

Our study results highlight the important role of miRNA dysregulation in the pathogenesis of reproductive disorders, including UI, PCOS, and PID. Our bioinformatics analysis revealed key miRNAs that were differentially expressed across these conditions, suggesting their potential as biomarkers for more accurate diagnosis. The involvement of central molecules, such as AGO2, AR, SMADs, or VIM, underscores the complex interplay between miRNA regulation, hormonal signaling, and tissue remodeling in these diseases. These findings lay the groundwork for insights into the molecular mechanisms driving reproductive disorders and emphasize the need for further research to develop targeted treatment strategies that may improve IVF success rates.

This study provides molecular-level evidence indicating that alternative criteria should be considered when evaluating IVF patients. In this regard, the initial step should involve a molecular characterization of the follicles based on criteria related to oocyte performance, such as the blastulation rate. This study provides molecular evidence suggesting the need for alternative evaluation criteria for IVF patients. A crucial first step would involve the molecular characterization of follicles using performance-based metrics, such as blastulation rates. Furthermore, these findings require additional validation in larger cohorts to confirm their reproducibility and reliability across diverse populations. Such large-scale studies would enhance the robustness of these molecular markers and support their integration into clinical practice, ultimately facilitating more personalized and effective treatments for infertility. One limitation of the present study is the relatively reduced sample size, which may restrict the generalizability of the results and highlights the need for further investigations involving more extensive patient populations.

## Figures and Tables

**Table 1:** Distribution of demographic and clinical factors and health-related behaviors among study participants.

Variable	N = Number of Subjects (%)
**Age (years)**	
**Mean ± SD**	35.2 ± 4.8
**Range**	26–44
**BMI (kg/m^2^)**	
**<18.5**	2 (4.3)
**18.5–25**	28 (59.6)
**25–30**	13 (27.6)
**≥30**	4 (8.5)
**Education**	
**College**	1 (2.1)
**Faculty**	36 (76.6)
**High school**	4 (8.5)
**Post high school**	4 (8.5)
**Secondary school**	1 (2.1)
**Vocational school**	1 (2.1)
**Infertility diagnosis**	
**PCOS**	5 (10.6)
**PID**	7 (14.9)
**Endometriosis**	3 (6.4)
**UI**	21 (44.7)
**Male factor**	9 (19.1)
**Mixed (female and male factor)**	2 (4.3)
**ICSI**	
**YES**	27 (57.4)
**NO**	20 (42.6)
**No. of fertilized oocytes**	
**Mean ± SD**	4.8 ± 2.8
**Range**	1–12
**No. of blastocytes**	
**Mean ± SD**	2.2 ± 1.4
**Range**	0–6
**Pregnancy**	
**YES**	20 (42.6)
**NO**	26 (55.3)
**Abortion**	1 (2.1)
**Smoking**	
***YES***	9 (19.1)
***NO***	38 (80.9)

BMI, body mass index; ICSI, intracytoplasmic sperm injection; SD, standard deviation.

**Table 2:** Study participants included in the microarray study.

	Male Factor Infertility Diagnosis	PCOS Diagnosis	PID Diagnosis	UI Diagnosis
Sample ID	C015	ID003	ID005	ID001
	C031	ID010	ID008	ID006
	C046	ID013	ID023	ID009
		ID016	ID026	ID012
		ID025	ID033	ID017
		ID028	ID036	ID018
		ID044	ID039	ID019
		ID045		ID024

**Table 3:** List of miRNAs with an altered expression level in study participants with PCOS, PID, or UI diagnosis versus a group of study participants with male factor infertility diagnosis.

PCOS			PID			UI		
miRNA	*p*-Value	FC	miRNA	*p*-Value	FC	miRNA	*p*-Value	FC
hsa-miR-5006-5p	0.048818	−2.26296	hsa-miR-320b	0.039884	−1.66046	hsa-miR-3652	0.032759	−1.5348632
hsa-miR-6785-5p	0.048797	−4.35155	hsa-miR-422a	0.030223	−2.08079	hsa-miR-423-5p	0.021881	−1.6792842
hsa-miR-3200-5p	0.041602	−6.29675	hsa-miR-4481	0.035729	−3.10321	hsa-miR-320a	0.010301	−1.6942542
hsa-miR-6800-3p	0.020221	−8.68356	hsa-miR-6785-5p	0.045551	−4.25992	hsa-miR-378a-3p	0.035554	−1.7705184
hsa-miR-1296-3p	0.021707	−8.71487	hsa-miR-3150b-3p	0.030758	−6.51924	hsa-miR-4651	0.032146	−1.9902356
hsa-miR-6743-5p	0.013994	−8.78707	hsa-miR-513a-3p	0.026792	−7.21145	hsa-miR-320b	0.003563	−2.05183
hsa-miR-7850-5p	0.006439	−9.30464	hsa-miR-5093	0.025999	−7.30568	hsa-miR-572	0.037317	−2.1064086
hsa-miR-8059	0.035159	−11.789	hsa-miR-8055	0.027552	−8.00561	hsa-miR-371b-5p	0.042022	−2.7818508
hsa-miR-96-5p	0.032253	−13.3004	hsa-miR-4715-5p	0.025002	−8.11684	hsa-miR-4481	0.037089	−4.7878485
hsa-miR-6753-3p	0.042607	12.67912	hsa-miR-3925-5p	0.027265	−8.56468	hsa-miR-6785-5p	0.02951	−5.4503827
hsa-miR-340-5p	0.046759	9.009871	hsa-miR-4440	0.035014	−9.58227	hsa-miR-1343-3p	0.02673	−5.9446187
hsa-miR-6864-3p	0.043599	5.416498	hsa-miR-1254	0.023006	−10.3288	hsa-miR-92b-3p	0.04412	−6.4570365
hsa-miR-29c-5p	0.037662	5.181044	hsa-miR-6817-5p	0.015223	−13.2246	hsa-miR-6817-5p	0.039526	−6.7536287
hsa-miR-4318	0.037197	5.16057	hsa-miR-7850-5p	1.91 × 10^−5^	−15.3778	hsa-miR-3180-5p	0.018344	−7.153791
hsa-miR-7-1-3p	0.036099	5.110514	hsa-miR-8059	1.37 × 10^−5^	−32.1081	hsa-miR-6800-3p	0.028772	−7.775571
hsa-miR-6825-3p	0.033382	4.972778	hsa-miR-4436b-3p	0.012431	15.05348	hsa-miR-6890-3p	0.038843	−9.462893
hsa-miR-6073	0.032759	4.937158	hsa-miR-4485-3p	0.020422	3.270453	hsa-miR-548am-5p	0.019529	−10.179389
hsa-miR-20a-3p	0.032314	4.91043	hsa-miR-494-3p	0.033067	2.803433	hsa-miR-3928-5p	0.013132	−10.692548
hsa-miR-4326	0.032242	4.906011	hsa-miR-6747-5p	0.044561	2.320432	hsa-miR-1470	0.02218	−11.310488
hsa-miR-15b-3p	0.028673	4.571666	hsa-miR-4644	0.008974	2.22851	hsa-miR-4440	0.006532	−11.646117
hsa-miR-301b-3p	0.028673	4.571666	hsa-miR-3135b	0.049657	1.9634	hsa-miR-1296-3p	0.004099	−11.660315
hsa-miR-545-3p	0.028673	4.571666				hsa-miR-7850-5p	0.004139	−12.055969
hsa-miR-93-3p	0.028673	4.571666				hsa-miR-1237-3p	0.013679	−12.200047
hsa-miR-494-3p	0.034822	4.073813				hsa-miR-4793-3p	0.00522	−13.793538
hsa-miR-8069	0.020086	2.061847				hsa-miR-4436b-3p	0.040373	10.764503
						hsa-miR-3164	0.014509	10.348244
						hsa-miR-663b	0.043826	5.7668953
						hsa-miR-513a-5p	0.014961	3.638524
						hsa-miR-513b-5p	0.036334	3.6029956
						hsa-miR-4485-3p	0.031918	2.792712
						hsa-miR-6747-5p	0.047025	2.4140615
						hsa-miR-4667-5p	0.048273	2.1719646
						hsa-miR-765	0.0452	2.1134443
						hsa-miR-1273e	0.048152	2.0018563

**Table 4:** Results of IPA analysis on altered miRNA patterns in the investigated study participants with PCOS, PID, or UI diagnoses, indicating the main biological processes targeted by altered miRNAs.

Biological Process	Group	Name	*p*-Value Range	# Molecules
diseases and disorders	PCOS	Organismal Injury and Abnormalities	4.61 × 10^−2^–4.06 × 10^−6^	10
		Reproductive System Disease	3.65 × 10^−2^–4.06 × 10^−6^	7
		Cancer	4.61 × 10^−2^–6.38 × 10^−4^	8
		Auditory Disease	9.06 × 10^−4^–9.06 × 10^−4^	1
		Hereditary Disorder	1.75 × 10^−2^–9.06 × 10^−4^	2
	PID	Inflammatory Disease	1.99 × 10^−2^–6.16 × 10^−6^	4
		Inflammatory Response	1.99 × 10^−2^–6.16 × 10^−6^	3
		Organismal Injury and Abnormalities	4.97 × 10^−2^–6.16 × 10^−6^	8
		Renal and Urological Disease	6.16 × 10^−6^–6.16 × 10^−6^	3
		Cancer	4.97 × 10^−2^–3.92 × 10^−5^	5
	UI	Organismal Injury and Abnormalities	4.57 × 10^−2^–2.14 × 10^−6^	15
		Psychological Disorders	3.32 × 10^−2^–2.14 × 10^−6^	8
		Inflammatory Disease	3.41 × 10^−2^–2.98 × 10^−6^	7
		Inflammatory Response	1.27 × 10^−3^–2.98 × 10^−6^	5
		Renal and Urological Disease	2.98 × 10^−6^–2.98 × 10^−6^	4
molecular and cellular function alteration	PCOS	Cellular Growth and Proliferation	3.13 × 10^−2^–1.61 × 10^−4^	8
		Cellular Movement	1.34 × 10^−2^–4.92 × 10^−4^	8
		Cell Death and Survival	2.38 × 10^−2^–9.57 × 10^−4^	3
		Cellular Development	3.13 × 10^−2^–1.36 × 10^−3^	6
		Cell Morphology	2.38 × 10^−2^–4.52 × 10^−3^	1
	UI	Cellular Movement	3.82 × 10^−2^–6.68 × 10^−4^	6
		Cell Death and Survival	4.17 × 10^−2^–3.33 × 10^−3^	3
		Cellular Development	3.76 × 10^−2^–3.57 × 10^−3^	5
		Cellular Function and Maintenance	9.85 × 10^−3^–3.57 × 10^−3^	3
		Cellular Growth and Proliferation	3.76 × 10^−2^–3.57 × 10^−3^	36
	UI	Cellular Movement	4.07 × 10^−2^–4.91 × 10^−4^	9
		Cellular Development	4.57 × 10^−2^–1.53 × 10^−3^	9
		Cellular Growth and Proliferation	4.57 × 10^−2^–1.53 × 10^−3^	9
		Cell-To-Cell Signaling and Interaction	1.73 × 10^−3^–1.73 × 10^−3^	1
		Cell Death and Survival	3.74 × 10^−2^–2.59 × 10^−3^	5

#: Number of the molecules.

## Data Availability

The raw data supporting the conclusions of this article will be made available by the authors on request.

## References

[1] Luke B. (2017). Pregnancy and birth outcomes in couples with infertility with and without assisted reproductive technology: With an emphasis on US population-based studies. Am. J. Obstet. Gynecol..

[2] Sabbagh R., Mulligan S., Shah J., Korkidakis A., Penzias A., Vaughan D., Patrizio P., Sakkas D. (2023). From oocytes to a live birth: Are we improving the biological efficiency?. Fertil. Steril..

[3] Ahmadi H., Aghebati-Maleki L., Rashidiani S., Csabai T., Nnaemeka O.B., Szekeres-Bartho J. (2023). Long-Term Effects of ART on the Health of the Offspring. Int. J. Mol. Sci..

[4] Wang B., Liu W., Liu Y., Zhang W., Ren C., Guan Y. (2021). What Does Unexpected Suboptimal Response During Ovarian Stimulation Suggest, an Overlooked Group?. Front. Endocrinol..

[5] Bosch E., Labarta E., Kolibianakis E., Rosen M., Meldrum D. (2016). Regimen of ovarian stimulation affects oocyte and therefore embryo quality. Fertil. Steril..

[6] Montano L., Raimondo S., Piscopo M., Ricciardi M., Guglielmino A., Chamayou S., Gentile R., Gentile M., Rapisarda P., Oliveri Conti G. (2025). First evidence of microplastics in human ovarian follicular fluid: An emerging threat to female fertility. Ecotoxicol. Environ. Saf..

[7] Adamczak R., Ukleja-Sokołowska N., Lis K., Dubiel M. (2021). Function of Follicular Cytokines: Roles Played during Maturation, Development and Implantation of Embryo. Medicina.

[8] Bianchi L., Gagliardi A., Landi C., Focarelli R., De Leo V., Luddi A., Bini L., Piomboni P. (2016). Protein pathways working in human follicular fluid: The future for tailored IVF?. Expert. Rev. Mol. Med..

[9] Braicu C., Calin G.A., Berindan-Neagoe I. (2013). MicroRNAs and cancer therapy—From bystanders to major players. Curr. Med. Chem..

[10] Braicu C., Catana C., Calin G.A., Berindan-Neagoe I. (2014). NCRNA combined therapy as future treatment option for cancer. Curr. Pharm. Des..

[11] Shekibi M., Heng S., Nie G. (2022). MicroRNAs in the Regulation of Endometrial Receptivity for Embryo Implantation. Int. J. Mol. Sci..

[12] Liang J., Wang S., Wang Z. (2017). Role of microRNAs in embryo implantation. Reprod. Biol. Endocrinol..

[13] Aoki S., Inoue Y., Hara S., Itou J., Shirasuna K., Iwata H. (2024). microRNAs associated with the quality of follicular fluids affect oocyte and early embryonic development. Reprod. Med. Biol..

[14] Qasemi M., Amidi F. (2020). Extracellular microRNA profiling in human follicular fluid: New biomarkers in female reproductive potential. J. Assist. Reprod. Genet..

[15] Redis R.S., Berindan-Neagoe I., Pop V.I., Calin G.A. (2012). Non-coding RNAs as theranostics in human cancers. J. Cell. Biochem..

[16] Cojocneanu R., Braicu C., Raduly L., Jurj A., Zanoaga O., Magdo L., Irimie A., Muresan M.S., Ionescu C., Grigorescu M. (2020). Plasma and Tissue Specific miRNA Expression Pattern and Functional Analysis Associated to Colorectal Cancer Patients. Cancers.

[17] Yang Y., Zhao C., Chen B., Yu X., Zhou Y., Ni D., Zhang X., Zhang J., Ling X., Zhang Z. (2023). Follicular fluid C3a-peptide promotes oocyte maturation through F-actin aggregation. BMC Biol..

[18] Chen B., Xu P., Wang J., Zhang C. (2019). The role of MiRNA in polycystic ovary syndrome (PCOS). Gene.

[19] Butler A.E., Ramachandran V., Sathyapalan T., David R., Gooderham N.J., Benurwar M., Dargham S.R., Hayat S., Hani Najafi-Shoushtari S., Atkin S.L. (2020). microRNA Expression in Women with and Without Polycystic Ovarian Syndrome Matched for Body Mass Index. Front. Endocrinol..

[20] Hon J.X., Wahab N.A., Karim A.K.A., Mokhtar N.M., Mokhtar M.H. (2023). MicroRNAs in Endometriosis: Insights into Inflammation and Progesterone Resistance. Int. J. Mol. Sci..

[21] Begum M.I.A., Chuan L., Hong S.T., Chae H.S. (2023). The Pathological Role of miRNAs in Endometriosis. Biomedicines.

[22] Neamtiu I.A., Surcel M., Begum T.F., Gurzau E.S., Berindan-Neagoe I., Braicu C., Rotar I., Muresan D., Bloom M.S. (2022). Specific lifestyle factors and in vitro fertilization outcomes in Romanian women: A pilot study. PeerJ.

[23] Zegers-Hochschild F., Adamson G.D., de Mouzon J., Ishihara O., Mansour R., Nygren K., Sullivan E., Vanderpoel S. (2009). International Committee for Monitoring Assisted Reproductive Technology (ICMART) and the World Health Organization (WHO) revised glossary of ART terminology, 2009. Fertil. Steril..

[24] Ciocan-Cartita C.A., Jurj A., Zanoaga O., Cojocneanu R., Pop L.A., Moldovan A., Moldovan C., Zimta A.A., Raduly L., Pop-Bica C. (2020). New insights in gene expression alteration as effect of doxorubicin drug resistance in triple negative breast cancer cells. J. Exp. Clin. Cancer Res..

[25] Roth L.W., McCallie B., Alvero R., Schoolcraft W.B., Minjarez D., Katz-Jaffe M.G. (2014). Altered microRNA and gene expression in the follicular fluid of women with polycystic ovary syndrome. J. Assist. Reprod. Genet..

[26] Sang Q., Yao Z., Wang H., Feng R., Wang H., Zhao X., Xing Q., Jin L., He L., Wu L. (2013). Identification of microRNAs in human follicular fluid: Characterization of microRNAs that govern steroidogenesis in vitro and are associated with polycystic ovary syndrome in vivo. J. Clin. Endocrinol. Metab..

[27] Santi D., Spaggiari G., Greco C., Lazzaretti C., Paradiso E., Casarini L., Potì F., Brigante G., Simoni M. (2021). The “Hitchhiker’s Guide to the Galaxy” of Endothelial Dysfunction Markers in Human Fertility. Int. J. Mol. Sci..

[28] Singh S., Pal N., Shubham S., Sarma D.K., Verma V., Marotta F., Kumar M. (2023). Polycystic Ovary Syndrome: Etiology, Current Management, and Future Therapeutics. J. Clin. Med..

[29] Fahs D., Salloum D., Nasrallah M., Ghazeeri G. (2023). Polycystic Ovary Syndrome: Pathophysiology and Controversies in Diagnosis. Diagnostics.

[30] Qin Y., Wang Y., Zhao H., Yang Z., Kang Y. (2021). Aberrant miRNA-mRNA regulatory network in polycystic ovary syndrome is associated with markers of insulin sensitivity and inflammation. Ann. Transl. Med..

[31] Yang Y., Lang P., Zhang X., Wu X., Cao S., Zhao C., Shen R., Ling X., Yang Y., Zhang J. (2023). Molecular characterization of extracellular vesicles derived from follicular fluid of women with and without PCOS: Integrating analysis of differential miRNAs and proteins reveals vital molecules involving in PCOS. J. Assist. Reprod. Genet..

[32] Huang J., Huang B., Kong Y., Yang Y., Tian C., Chen L., Liao Y., Ma L. (2022). Polycystic ovary syndrome: Identification of novel and hub biomarkers in the autophagy-associated mRNA-miRNA-lncRNA network. Front. Endocrinol..

[33] Palagiano A., Cozzolino M., Ubaldi F.M., Palagiano C., Coccia M.E. (2021). Effects of Hydrosalpinx on Endometrial Implantation Failures: Evaluating Salpingectomy in Women Undergoing in vitro fertilization. Rev. Bras. Ginecol. Obstet..

[34] Ng K.Y.B., Cheong Y. (2019). Hydrosalpinx—Salpingostomy, salpingectomy or tubal occlusion. Best Pract. Res. Clin. Obstet. Gynaecol..

[35] Batushansky A., Zacharia A., Shehadeh A., Bruck-Haimson R., Saidemberg D., Kogan N.M., Thomas Mannully C., Herzberg S., Ben-Meir A., Moussaieff A. (2020). A Shift in Glycerolipid Metabolism Defines the Follicular Fluid of IVF Patients with Unexplained Infertility. Biomolecules.

[36] Vaigauskaitė-Mažeikienė B., Baušytė R., Valatkaitė E., Maželytė R., Kazėnaitė E., Ramašauskaitė D., Navakauskienė R. (2023). Assisted reproductive technology outcomes and gene expression in unexplained infertility patients. Front. Cell Dev. Biol..

[37] Müller M., Fazi F., Ciaudo C. (2019). Argonaute Proteins: From Structure to Function in Development and Pathological Cell Fate Determination. Front. Cell Dev. Biol..

[38] Kusumaningtyas I., Dasuki D., Harjana S.M., Sadewa A.H., Sweety M.C., Septiani L. (2024). Unraveling the microRNAs, key players in folliculogenesis and ovarian diseases. Middle East Fertil. Soc. J..

[39] Yoo J.Y., Ku B.J., Kim T.H., Il Ahn J., Ahn J.Y., Yang W.S., Lim J.M., Taketo M.M., Shin J.H., Jeong J.W. (2020). β-catenin activates TGF-β-induced epithelial-mesenchymal transition in adenomyosis. Exp. Mol. Med..

[40] Kobayashi H., Kishi Y., Matsubara S. (2019). Mechanisms Underlying Adenomyosis-Related Fibrogenesis. Gynecol. Obstet. Investig..

[41] Walters K.A., Rodriguez Paris V., Aflatounian A., Handelsman D.J. (2019). Androgens and ovarian function: Translation from basic discovery research to clinical impact. J. Endocrinol..

[42] Wang K., Li Y., Chen Y. (2023). Androgen excess: A hallmark of polycystic ovary syndrome. Front. Endocrinol..

[43] Ostrowska-Podhorodecka Z., Ding I., Norouzi M., McCulloch C.A. (2022). Impact of Vimentin on Regulation of Cell Signaling and Matrix Remodeling. Front. Cell Dev. Biol..

